# The Role of Multistakeholder Platforms in Environmental Governance: Analyzing Stakeholder Perceptions in Kalomo District, Zambia, Using Q-Method

**DOI:** 10.1007/s00267-023-01806-z

**Published:** 2023-03-20

**Authors:** Freddie S. Siangulube

**Affiliations:** 1 Amsterdam Institute for Social Science Research (AISSR), University of Amsterdam, Nieuwe Achtergracht 166, 1018 VW Amsterdam, The Netherlands; 2 Centre for International Forestry Research (CIFOR), Bogor, Indonesia

**Keywords:** Multistakeholder platforms, Narratives, Discourses, Landscape challenges, Stakeholder perspectives, Q-method, Kalomo District, Zambia

## Abstract

Multistakeholder platforms (MSPs) are increasingly applied in environmental governance as institutions to collectively negotiate challenges, opportunities, and policy options in contested landscapes. However, their contributions and effectiveness depend on how stakeholders perceive and frame the role of MSPs in addressing social and environmental challenges. Despite this dependence, stakeholder perceptions of MSPs are currently under-researched. Hence this empirical study carried out in Zambia’s Kalomo District asks: how do stakeholder groups perceive the role of MSPs in addressing landscape challenges, given the context of the dual land tenure system, and what does this imply for the implementation of integrated landscape approaches? This study uses Q-methodology to analyze the perceptions of purposefully selected stakeholders from state institutions, civil society organizations, land users, and others familiar with existing MSPs at the district and village levels. The findings reveal three narratives. The first one presents MSPs as institutions that foster dialogue. The second narrative foregrounds the role of the government and private sector, despite acknowledging the diversity of stakeholders in MSPs. In this narrative, MSPs should focus on supporting market-driven solutions to resolve landscape challenges. The third narrative recognizes power imbalances and considers MSPs as institutions to identify policy gaps and needs. The first two narratives are positioned in Dryzek’s discourse classification as environmental problem-solving, while the third inclines toward green radicalism. Despite this divergence, there was consensus that MSPs have the potential to harmonize policies in a dual governance system and encourage dialogue between stakeholders to reconcile landscape challenges.

## Introduction

The contribution of multistakeholder platforms (MSPs) in reconciling conflicting objectives has gained increasing attention in contemporary global environmental governance. This is particularly the case for ‘wicked problems’ such as food security and climate change (Ray et al. 2019), unsustainable and responsible consumerism (Geels et al. 2015), reconciling development and biodiversity conservation (Jeffrey 2009), and harmonizing targets within the Sustainable Development Goals agenda (Fowler and Biekart 2017). Some international and national civil society organizations, as well as local communities lacking the power to participate in national and global governance affairs, consider MSPs as a potentially legitimate mechanism for engagement (Gleckman 2018). At the global level, there is progress in embedding “multistakeholderisms” in governance systems (Zanella et al. 2018), such as the Nationally Determined Contributions partnership in the United Nations system (Larson et al. 2022).

In the global South, MSPs are increasingly endorsed for their potential to contribute to research and innovation (Hermans et al. 2017), influence inclusive policy processes in natural resource management and livelihoods (Warner 2006; Sartas et al. 2018), and facilitate dialogue among conflicting interest groups (Kusters et al. 2020). Like most sub-Saharan African regions, in Zambia an array of MSP types exist at various jurisdictional levels as either formal or informal governance institutions. For most MSPs with an environmental governance focus, their forms and functions vary based on the contextual landscape issues participants are confronted with. These institutions typically engage diverse interest groups in participatory and inclusive dialogue toward negotiating trade-offs between different land-uses. This paper refers to these groups as stakeholders, defined as those who have an interest in the landscape and are affected favorably or adversely by the actions of others or whose actions affect others (Dale et al. 2019). For instance, MSPs in the Kalomo District of Zambia are intertwined in governance arrangements between traditional and state regulations, placing them central to facilitating dialogue-focused solutions to land management issues. These MSPs are often convened through established statutory mechanisms and directed by the heads of government institutions. At the local level, traditional leaders with the delegated authority of the village chief are responsible for convening stakeholders, depending on the issues at hand.

While MSPs are recognized as pathways for participatory governance in which policy decisions are cognizant of the voices of marginalized people (Sarmiento-Barletti and Larson 2019; Larson et al. 2022), some scholars question this romanticized assumption for a variety of reasons (McKeon 2017; Metzger et al. 2017). For instance, McKeon (2017, p. 379) argues that the acclaimed “win-win affairs” in MSPs lack validation by empirical research, particularly in platforms in which private and public interests converge. Others argue that the potential of MSPs in many natural resource management domains is rather speculative and overlooks the effects of platform composition (Faysse 2006) and power imbalances mostly because measuring MSP effectiveness is influenced by stakeholders’ expectations and perspectives of what MSPs should accomplish (Sayer et al. 2017).

Given this background, it may be argued that various discourses regarding the roles of MSPs exist. However, in landscape governance literature, discourses about how stakeholders perceive and frame the role of MSPs in addressing landscape challenges have received limited attention (Sarmiento Barletti et al. 2022). Nevertheless, MSPs are widely promoted as an essential component of integrated landscape approaches (ILAs) that aim to bring stakeholders together to negotiate trade-offs between competing land uses and conservation-development aims (Kusters et al. 2020; Ratner et al. 2022). Despite this, there is insufficient literature examining how stakeholders perceive the role and function of MSPs to help resolve social-ecological challenges at the landscape level (Larson et al. 2022). To address this research gap, I examined stakeholders’ perceptions of existing MSPs (at the district and village levels) aimed at finding solutions to land-use concerns in southern Zambia’s Kalomo District. Given the land tenure system in Kalomo that incorporates both state and customary governance systems, land-use conflicts are prevalent, and this theme has been a dominant discourse in MSPs. In this paper, I contend that understanding stakeholders’ wide spectrum of perspectives may enhance the efficacy of MSPs in similar tropical contexts, uncover more inclusive pathways toward landscape governance, and provide guidance that could support the implementation and sustainability of ILAs.

This paper addresses the following research question: How do stakeholders in Zambia’s Kalomo District perceive the role of MSPs in addressing landscape challenges, and what does this imply for the implementation of landscape approaches? I used the Q-methodology, a semi-quantitative approach, to unravel stakeholders’ divergent perspectives.

## Conceptual Background: Framing Discourses on MSPs

Gregory Bateson’s “A theory of play and fantasy” (Bateson [1954] 1972) sets the tone for the concept of framing, which has influenced the language of discourses and perceptions in many disciplines. The framing theory is predicated on the understanding that an issue can be interpreted and constructed from different perspectives and has implications for people who consider multiple alternatives (Chong and Druckman 2007). It refers to the cycle of processes by which humans create specific conceptions of an issue to help refocus their thinking about a problem (Tannen 1993). For the conceptual footing of stakeholder perceptions about MSPs, I build on Whyte’s (1977) and Mani-Peres et al.’s (2016) environmental perceptions, described as awareness and interpretation of the environment. In this study, stakeholder perceptions refer to how people view the material world by interpreting their experiences and expectations. Thus, in the language of Dryzek and Niemeyer (2008), perceptions are associated with discursive formations about the problems aimed at finding solutions. Discourses about MSPs, like most discursive platforms, are essentially a collection of perceptions about the human-environment relationship communicated through language that explains their material reality (Carpentier et al. 2021).

Scholars have applied different terms to refer to MSPs in the landscape governance literature, including stakeholder forums, multistakeholder dialogues, stakeholder partnerships, and multistakeholder networks (Barletti and Larson 2019; van Ewijk and Ros-Tonen 2021; Ratner et al. 2022). Often, stakeholder perceptions are reflected in how MSPs are defined and what they are expected to accomplish. In the recent decade, the roles of MSPs in landscape governance have become more recognized for their ability to support participatory and inclusive decision-making (Ratner et al. 2022). Landscape governance approaches such as ILAs that seek to reconcile divergent interests require a fuller appreciation of the differentiated opinions of diverse stakeholders. By doing so, governance approaches gain new and more inclusive insights, thus enabling continual learning and adaptive management (Sayer et al. 2013).

**Fig. 1:**
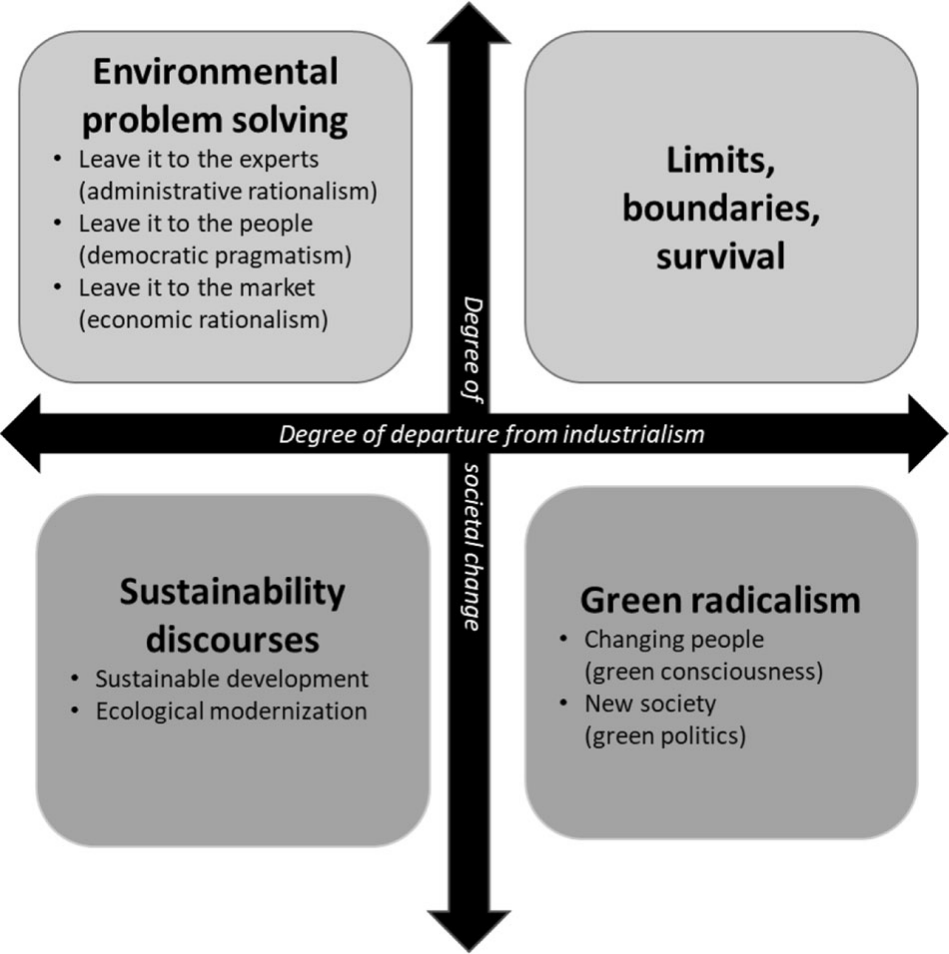
Classification of environmental discourses. Source: Dryzek 2013

The presence of multiple stakeholders at the landscape level entails dealing with various perspectives or framings. Reed et al. (2022) showed how negotiating a theory of change^1^ through MSPs revealed discourses that helped uncover dynamics (e.g., drivers of environmental dynamics) in a tropical landscape—highlighting how human expectations, experiences, and interactions with nature shape stakeholder perceptions of common concerns, consistent with the theory of normative expectations and attitudes (Hjortskov 2019). Recognizing the importance of negotiating collective goals through MSPs has prompted a research focus on MSPs themselves with perspectives such as environmental justice, land rights and access to natural resources, and landscape sustainability. However, such an assemblage of perspectives is reflected in several discursive framings. Dryzek (2013) distinguishes four main discourse groups (Fig. 1) based on two dimensions: the degree of departure from the commitment to economic growth (reformist versus radical) and the degree of societal change (prosaic versus imaginative). Environmental problem-solving discourses are reformist-prosaic; the limits, boundaries, and survival discourse is radical-prosaic; sustainability discourses are reformist-imaginative, and green radicalism is classed as radical-imaginative. Each of these main discourses has its own sub-discourses. This classification informed the interpretation of the various perspectives examined in this paper.

There are four reasons why insights into discourse framing are necessary for achieving inclusive stakeholder-centered strategies such as ILAs. First, unraveling the conceptual understanding of the functions of MSPs across stakeholder groups helps identify the basis for distilling “common concern entry points” and “negotiated and transparent change logic” (Sayer et al. 2013). Second, acknowledging that no single rationality or objective truth may claim to define the ideal MSP is critical to embracing the plurality of stakeholders and their viewpoints, which adds new dimensions to problem analysis and facilitates “continuous learning and adaptive management” (Sayer et al. 2013, p. 8351). Third, a collection of discourses about MSPs may contain complementary or competing views necessary to enrich policy options from different perspectives (Ratner et al. 2019). This array of perspectives enriches debates in developing research-informed policies for landscape sustainability. Fourth, unraveling discourses lays the basis for identifying potential stakeholder coalitions (Newig et al. 2010).

## Methodology

### Background to the Study Area

**Fig. 2:**
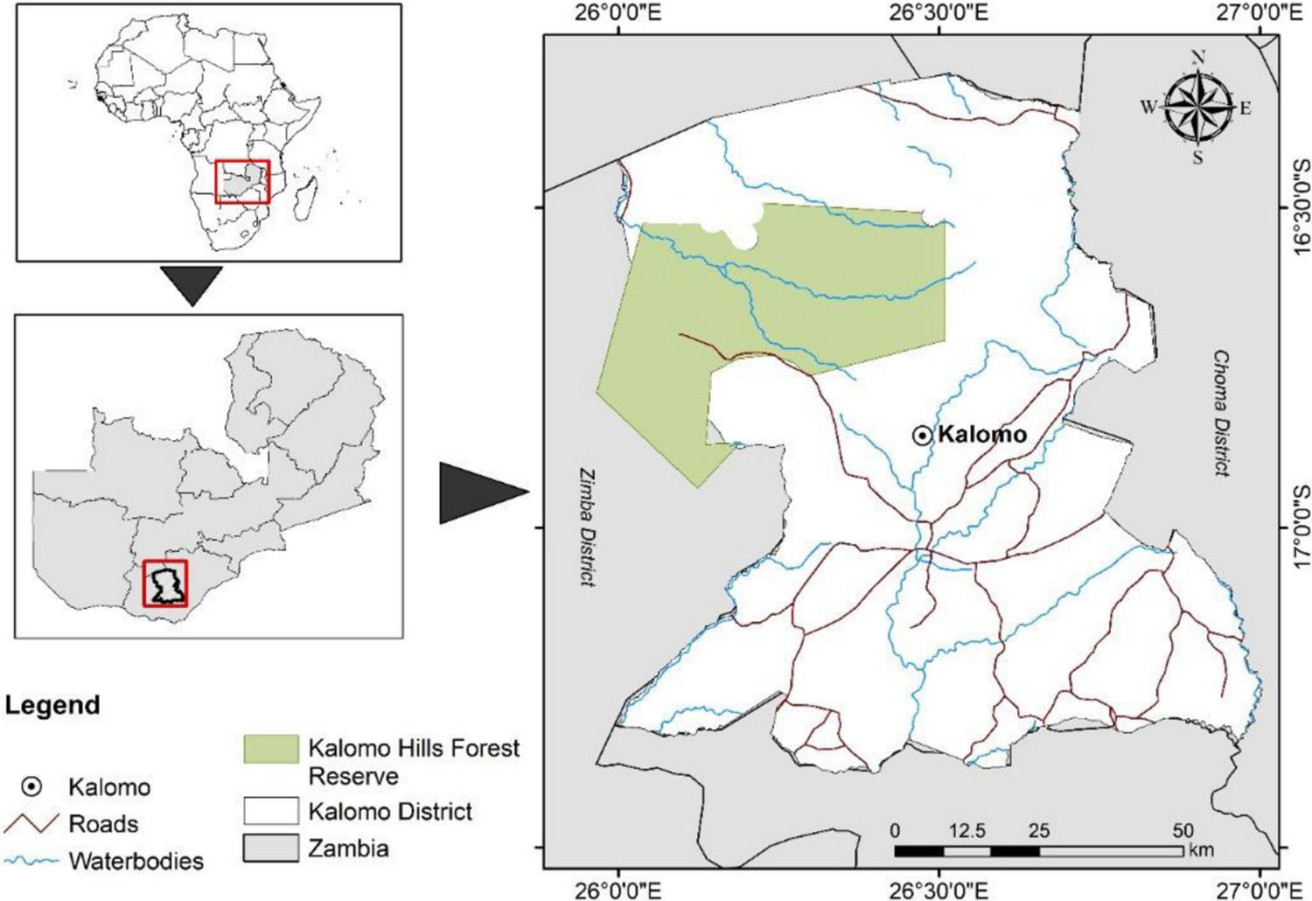
Location map of the study area, the Kalomo District (Source: Siangulube et al. 2023)

The study area corresponds with the operational area of the COLANDS^2^ initiative that seeks to implement ILAs in Zambia’s Kalomo District (Fig. 2). Kalomo District (alternately referred to as the Kalomo landscape), with a population of over 170,000 people, is the largest district in Zambia’s Southern Province and lies in a tourism-agriculture-conservation corridor (Moombe et al. 2020). The landscape is home to the province’s largest forest reserve and is bordered on the west by the famed second-largest protected game park in Africa, the Kafue National Park (Thapa 2012), and on the south by the Sichifulo Game Management Area. The Kalomo landscape is ~130 kilometers north of the Victoria Falls World Heritage Site, one of the Seven Wonders of the World (Dube and Nhamo 2019), and remains one of the main regional tourism destinations. The intersection of conservation, development, and agricultural land uses presents a substantial challenge in terms of landscape management and decision-making (Stone et al. 2022). In recent decades, the increasing demand for food production and other ecosystem services has largely influenced the economic dynamics and local livelihoods, putting pressure on land resources, notably water, grazing land, and forests (Moombe et al. 2020; Upla et al. 2022). Although no specific data is available for Kalomo District, the overall rural Gini coefficient of 0.61 indicates severe social inequality (Reed et al. 2020).

Consistent with other parts of Zambia, landscape governance at the district and sub-district levels is associated with the dual land tenure system that influences access and restrictions to land resources (Chilombo 2021). This dual land tenure system in Kalomo District constitutes customary and statutory tenure systems. In customary land tenure, which has existed since the pre-colonial period (van Loenen 1999), the chief regulates land access, rights, and allocation on behalf of communities through various sub-chief institutions. Statutory governance is administered through the Ministry of Lands and Natural Resources and other supporting central government institutions such as the Forestry Department.

The interaction between these two concurrent land systems in the Kalomo landscape is complex and has often exacerbated land-use conflicts and, in some ways, perpetuated the marginalization of poor farmers, especially around the Kalomo Hills forest reserve, where boundary disputes remain unresolved (Moombe et al. 2020). Historically, the development of the statutory system in the study area follows regional patterns. Shifting away from customary to statutory land governance was ‘glorified’ by the World Bank’s land reform agenda in most African countries in the 1980s (Byamugisha 2014) and is based on a neoclassical economic model that claims that property registration encourages investment in land, makes available better credits, and improves efficient land markets, resulting in sustainable agricultural outputs and a well-managed landscape (Smith 2005). However, recent research in the Kalomo landscape indicates that the transition has instead had unintended detrimental consequences on land management (Muchimba 2022), including aggravating marginalized farmers’ already fragile tenure security and widening the rich-poor divide. Various village and district-level MSPs are embedded in these governance layers that interact in complementary and, in some cases, conflicting ways of dealing with landscape challenges. (see Appendix 1 in the Supplementary Material that shows the orientational strands of MSPs in the Kalomo District based on interviews with research participants). At the district level, the District Development Coordinating Committee is the main formal MSP alongside others, such as the District Consultative Group and the Constituency Development Committee. At the sub-district level, several MSPs exist. Depending on the form and the nature of the problems, Village Productivity Committees prominently endeavor to address land-use challenges.

### The Q-methodology Research Design

I applied the Q-methodology to elicit various stakeholder perspectives on the potential of MSPs to mediate landscape challenges. Introduced by Stephenson in the 1930s, the Q-methodology is used to identify shared attitudes, perceptions, and preferences among groups on a specific subject (Stephenson 1935). In the Q-methodology procedure, respondents rank various statements on a quasi-normal distribution grid that looks like a pyramid (Zabala and Pascual 2016 see Appendix 2 in the Supplementary Material). The rankings on the grid range from “most agree” to “most disagree” to answer a predetermined study question (Zabala and Pascual 2016). In this case, the question posed on a card to the respondents was: “*What is the potential of MSPs to address landscape challenges in Kalomo District?”*. The term Q-methodology refers to a type of factor analysis in which subjects are grouped or clustered based on commonalities (Stephenson 1935; Langston et al. 2019a; Biersteker et al. 2022). This methodology is exploratory and semi-quantitative (combines qualitative data collection and quantitative factor analysis) and provides logical and systematic means of elicitation of multiple perspectives from many stakeholders (Zabala et al. 2018).

Scholars have applied the Q-methodology for studying human subjectivity on a specific topic (Watts 2015), including in conservation and environmental domains (Zabala et al. 2018; Ihemezie et al. 2022) and in framing ecological sustainability discourses (e.g., Barry and Proops 1999). The Q-methodology is appropriate for the study reported in this paper because it focuses on stakeholders’ holistic narratives of a problem (Watts and Stenner 2012), i.e., human behavior, perceptions in the real world, or the construct of MSPs to deal with landscape challenges.

### Data Collection

I followed the standard key steps to collect data that included: (i) concourse development, which entails identifying the spectrum of contemporary discourses supporting the potential of MSPs to address landscape problems; (ii) formulation of the Q-sort consisting of proxy statements; (iii) selection of the P-set, that is the respondents to be involved in the sorting exercise; (iv) Q-sorting of statements between “Most agreed” to “Least agreed”; and “Most disagreed to Least disagreed” and post-sorting interviews; (v) data analysis that involves factor extraction, factor rotation, and flagging and interpretation of factors (Watts 2015; Langston et al. 2019).

I included as many opinions as possible from various stakeholder groups, obtained through what Biersteker et al. (2022) refer to as exploratory conversations with knowledgeable persons or experts on the topic. The information for concourse development had to convey the theoretical concepts of MSP functionality and reflect the basis for understanding respondents’ discourse framings in everyday conversations (Brown 1993). Initially, information for concourse development was collected through a face-to-face trial exercise with eight diverse people conducted during an informal side meeting at a participatory theory of change workshop organized by the COLANDS initiative. The workshop discussed landscape challenges in the Kalomo District and drew participants from various backgrounds (see Reed et al. 2022). Additional statements for concourse development were obtained from the workshop proceedings and various literature. Later, other opinions on the subject were collected through open-ended interviews with target experts and cross-checked with local MSP participants (Table 1). Finally, two independent expert scholars verified the statements to ensure validity, clarity, and diversity were captured. This array of sources of information was gathered to ensure that the Q-sort was as broad and diverse as possible.

**Table 1:** Sources of information to decipher opinions for concourse development

Sources of information	Expertise or knowledge
Persons from local communities, government, and civil society organizations	Knowledge of functionalities of various MSPs or participants affiliated with one or two MSPs.
Researchers	Experience in facilitating or participating in MSP dialogues and a good knowledge of land uses and contestations in the study area.
Other sources	Literature, cross-checking with other people at local-level MSPs, general statements from the theory of change workshop proceedings, open-ended interviews with experts, and opinions from scholars.

Source: The Author

A critical step in the Q-methodology is identifying appropriate statements from the concourse called the Q-set (Hermans et al. 2012). There are two basic approaches to assessing concourse statements to generate a manageable Q-set—structured and unstructured (Hermans et al. 2012). The topic of MSP is very wide, and for this study, I used the structured sampling technique to create a broad-based Q-set constructed on Dentoni et al. (2018) analytical framework, which conceptualizes MSP governance in terms of deliberation, inclusive decision-making, and legitimacy. This approach helped to focus the analysis on MSP governance issues while at the same time ensuring that the Q-set was sufficiently divergent to represent the subjective diversity of all potential viewpoints.

I identified 66 broad statements, and after further scrutiny and refinement, redundancies were eliminated, especially where statements conveyed similar information. Eventually, 45 statements were retained while preserving the diversity of perspectives on the topic. The statements were carefully rephrased, simplified, and clarified to reflect relevance to the Kalomo landscape. A trial Q-sort interview was conducted with three persons (from an NGO, a village MSP, and a government official), culminating in further adjustments to improve clarity and data validity. Finally, a Q-sort with 42 statements was produced that focused on three main themes: land uses related to development and conservation (*n* = 12), conflict management (*n* = 15), and governance and funding issues (*n* = 15) (Table 3). This number of statements was deemed sufficient for a person to sort with concentration. According to Zabala and Pascual (2016), the number of statements should be between 40 and 80.

A comprehensive systematic inventory of prospective stakeholders in Kalomo District was conducted. The criteria for selecting participants, called the P-set, focused on people with relevant knowledge and experience dealing with natural resource challenges at the landscape level through the district- or local-level MSPs in Kalomo District (Velde et al. 2019). An underlying assumption in the Q approach is that “only a limited number of perspectives on any given topic exist within a group of people; implying that people are consistent and coherent in their perspectives, and it is thus likely that people of a particular mindset think about distinct issues in consistent ways” (Buckwell et al. 2020, p.4). Watts (2015) recommends fewer participants (P-set) than the number of statements in the Q-set. An additional criterion was that participants should represent sector domains related to natural resource conservation, governance, and development. Table 2 presents the stakeholder categorization in Q-sorting (interviews). Based on the criteria above, two individuals were initially selected as participants who had previously moderated district and local MSP meetings, respectively. One of these two was a village member with many years of experience moderating land-use conflicts in village-level MSPs, assumed to represent local-level perspectives. Then, using a non-probability snowball selection method, suitable participants meeting the earlier mentioned criteria were added based on recommendations. These were contacted for a short pre-Q-sort conversation to ascertain their suitability. Finally, 25 people consented to participate in the Q-methodology process (Table 2). To capture different perspectives, the main variable hinged on sector representativeness and not the sample size of the population (Zabala and Pascual 2016).

**Table 2:** The categories of Q-participants

Category^a^	Definition	No. of respondents (*n*)
Stakeholders from state institutions	Government stakeholders with state-related interests and influence on the implementation of national policies relating to natural resource management, conservation, governance, and livelihoods. This category included heads of government departments and the district commissioners’ office.	8
Civil society organizations (CSOs)	CSOs included non-state stakeholders whose aims are neither to generate profits nor to seek governing power. CSOs foster governance and the rights of people to advance shared goals and interests. This category included national NGOs, faith-based organizations, and local community-based organizations (CBOs, including women’s groups).	6
Local leaders	Local governance and leadership involved in administrating resources, land, and other village-level governance matters. These include chiefs’ representatives and village head persons.	4
Private sector	Stakeholders involved in enterprise development, trade, and commercially providing goods and services. These include seed companies, local entrepreneurs, tobacco companies, and charcoal producers.	3
Researchers	Stakeholders providing research, knowledge, and information on landscape governance issues, including social-ecological issues and landscape impacts and vulnerabilities.	2
Development/funding/others	Stakeholders championing a development agenda, including a representative of a local organization that draws membership from the government, private sector, local leadership, and a donor.	1
Village member	An inhabitant who has resided in a village for at least 2 years, helped coordinate MSPs for a long time, and is assumed to know the landscape challenges to represent village-level perspectives.	1
Total		25

^a^Names withheld to guarantee anonymity

At the beginning of the Q-sorting exercise, participants were guided to read and understand all the statements. To commence the sorting, each participant had to separate statements into three categories either “agreed”, “neutral” (neither agree nor disagree), or “disagreed”. Participants then placed the statements on a grid (see Appendix 2 in the Supplementary Material). Participants could re-order the statements as often as they wanted until they were satisfied. This exercise lasted between 1 and 1½ hours. A post-grid sorting interview was later conducted to seek clarification on the reasons participants gave for how they organized their statements (Ihemezie et al. 2022).

### Q-analysis

Ken-Q Analysis software (version 0.11.1) was used to analyze the Q sorts, build the 25 × 25 correlation matrices and extract factors. The aim of factor analysis is to identify the heterogeneity of participants’ views and categorize them based on shared views, referred to as factors. Each factor represents a particular perspective. To extract factors from the dataset, principal component analysis was utilized because it mathematically shows how each variable is related to the others (covariance matrix), the directions in which information is spread (eigenvectors), and the relative significance of factor directions (eigenvalue) (Brown 2015; Armatas et al. 2017; Mahlalela et al. 2022).

The initial principal component analysis returned eight factors that were then rotated with the Varimax technique to ensure that the appropriate factors accounted for the greatest amount of variance. The goal is to assign each variable to no more than one factor. It rotates all the factors as a group and centers on the largest group. As a common practice in Q-analysis, the decision criteria for selecting the number of factors followed the Kaiser-Guttman criterion, which recommends that only factors with an eigenvalue of 1.00 or higher and factors that have at least two Q sorts loading significantly on the factor should be retained (Watts and Stenner 2012) and Humphrey’s rule, which states that a factor is significant if the cross-product of its two highest loadings (ignoring signs) exceeds twice the standard error (Brown 1993).

Applying these decision criteria, factors 4–8 were excluded. As a result, three factors were isolated and rotated once more (see factor matrix with defining Q-sorts in Appendix 3 in the Supplementary Material). Factor rotation improves factor interpretability by making it possible to identify the optimal rotation for each factor’s load of statements (Zabala and Pascual 2016). Later, I investigated the correlation between factor arrays and discovered that none of the three factors was significantly positively associated with each other (*p* < 0.01), meaning I could concentrate on a three-factor solution for interpretation and conclusion.

### Dealing with Possible Biases

**Fig. 3:**
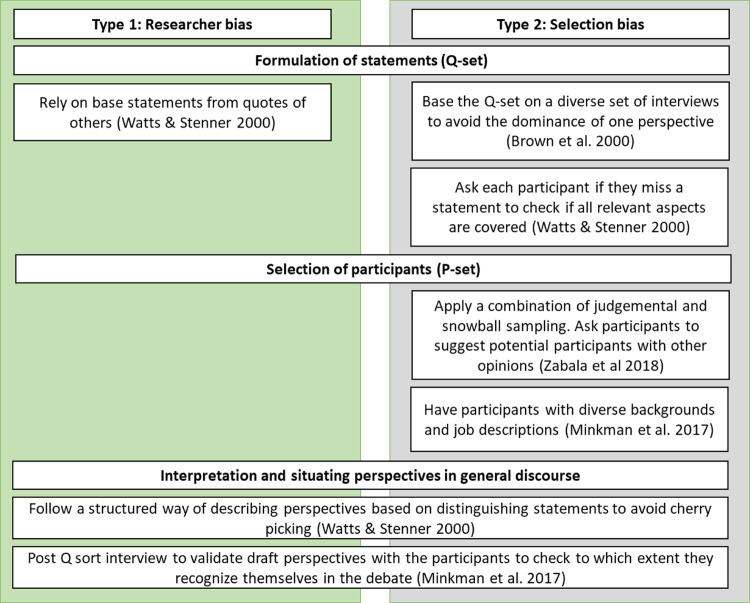
Approach to eliminate researcher bias (left) and selection bias (right). *Source:* modified from Minkman et al. 2017

The critics of using the Q-methodology to study human subjectivity raise validity issues arising from various biases (Kampen and Tamás 2014). In this study, there could have been two sources of data bias: researcher and selection biases. First, when the formulation of statements (Q-set) is overly dependent on the researcher’s opinions and knowledge, this could lead to researcher bias. Second, in non-random surveys, an error may occur in selecting (P-set) participants skewed toward a specific group, thus excluding other potential respondents (Minkman et al. 2017). Since the sample is not representative of the population, the number of participants is less crucial than ensuring that the key discourses being studied are included (Brown 1993). Figure 3 depicts some of the strategies applied in this study to eliminate biases.

## Results

The analysis identified three factors from the initial 42 Q-sorts. A varimax rotation flagged 25 Q-sorts on the three factors (SM I). Other statements with a flagging frequency lower than 0.6 were excluded because they contained ambiguous information to allow meaningful analysis (Mahlalela et al. 2022). Based on distinguishing statements, factor number one represents most of the explained variability with 13 Q-sorts, accounting for 42% of the variance. This was labeled *democratic institutions for equitable and inclusive decision-making*. The second factor, *forums that support market-based solutions committed to sustainable growth*, covered eight Q-sorts, representing 24% of the variation. The third factor, *addressing power imbalance and identifying policy gaps and needs*, summarized four Q-sorts and represented 13% of the variance.

In this study, the cumulative variance of 79% (42 + 24 + 13) is higher than the common occurrence in some Q-studies. However, in studying subjectivity on an issue whose scope is specific such as understanding the roles of MSPs in addressing landscape challenges in a Kalomo landscape, not so many divisive perspectives are expected; hence, a higher cumulative variance is seen in a few factors. Similarly, some studies, such as Gruber (2011), have documented a cumulative variance of 53% in a few factors. Summak and Kalman (2020) recorded 50% of the variance explained by a single factor in an extreme case.

**Fig. 4:**
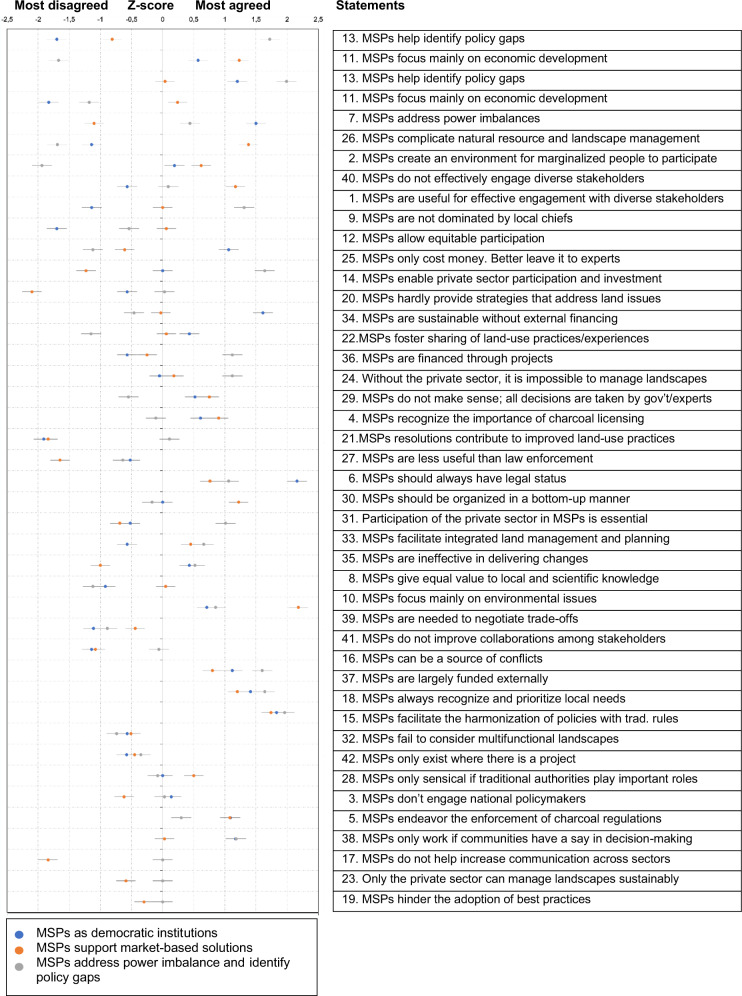
Q-statements (in some cases shortened to fit the cells) based on *Z*-score differences for participants’ perceptions about MSPs. Statements are arranged with the most distinguishing on top and consensus statements on the bottom. (Note: due to overlaps, only one dot is displayed when a statement has the same *Z*-score from two factors. For *z*-scores, see Appendix 5 in Supplementary Material)

Figure 4 shows the *Z*-scores for each statement. A *Z*-score value describes the position of each statement in the Q-sort table. The higher the *Z*-score value, the more extreme (positive or negative) its position is increasingly toward the edge of the Q-sort table, and this is the most useful statement in interpreting factors. As shown in Fig. 4, the wider the spread among the *Z*-scores (wider spread statement on the top) entails, the more disagreement in that statement, especially if that spread crosses the agree/disagree line. Conversely, the more *Z*-scores cluster, the more likely there is consensus in that statement (consensus statement toward the bottom). The Q-sort with *Z*-scores and ranks in all the factors (see Appendix 4 in the Supplementary Material) shows the order of importance and position of a statement in a factor. The following sub-section presents the different stakeholder perspectives.

### Stakeholder Perspectives

This section presents three perspectives based on the dominant perspectives from the Q-sort analysis and additional insights from post-Q-sort interviews. The first one presents MSPs as institutions that foster dialogue. The second narrative foregrounds the role of the government and private sector in which the narratives position MSPs as institutions that should focus on supporting market-driven solutions to resolve challenges. The third narrative recognizes power imbalances and considers MSPs as brokers to identify policy gaps and needs. The first two narratives are positioned in Dryzek’s discourse classification as environmental problem-solving (democratic pragmatism and administrative and economic rationalism, respectively), while the third narrative inclines toward a green radicalism (green politics) discourse.

#### Perspective 1: MSPs as democratic institutions for equitable and inclusive decision-making

The respondents adhering to this discourse framing were very heterogeneous. They include representatives of various backgrounds: community-based organizations (two women’s groups and an affiliate of a local faith-based organization, among others), traditional leaders, researchers, and private sector stakeholders. The information between brackets in the factor descriptions below pertains to the statement number (#) in Table 3.

**Table 3:** Q-sort with corresponding factors

Statement No.	Statements	Factor 1	Factor 2	Factor 3
1	MSPs are useful for effective engagement with diverse stakeholders	0	1	−4
2	MSPs create an environment for marginalized people to participate	3	−2	1
3	MSPs don’t engage national policymakers	0	−1	0
4	MSPs recognize the importance of charcoal licensing	1	1	−1
5	MSPs endeavor to facilitate the enforcement of charcoal regulations	2	1	1
6	MSPs should always have legal status	−1	−1	−3
7	MSPs address power imbalances	2	0	4
8	MSPs give equal value to local and scientific knowledge	1	−2	1
9	MSPs are not dominated by local chiefs	−1	2	1
10	MSPs focus mainly on environmental issues	−2	0	−2
11	MSPs focus mainly on economic development	1	3	−3
12	MSPs allow equitable participation	−2	0	−2
13	MSPs help identify policy gaps	−3	−2	3
14	MSPs enable private sector participation and investment	2	−1	−2
15	MSPs facilitate the harmonization of policies and laws with traditional customs and regulations	2	2	2
16	MSPs can be a source of conflicts	−2	−2	0
17	MSPs do not help increase communication across sectors	0	−3	0
18	MSPs always recognize and prioritize local needs	3	2	3
19	MSPs hinder the adoption of best practices	0	0	0
20	MSPs hardly provide strategies that address land issues, e.g., conflict over use	0	−2	3
21	MSPs resolutions contribute to improved land-use practices	1	2	0
22	MSPs foster sharing of land-use practices/ experiences	3	0	−1
23	Only the private sector has the innovative capacity to manage landscape sustainability	0	−1	0
24	Without the private sector, it is impossible to manage landscapes sustainably	−1	0	2
25	MSPs only cost money. Better leave it to government agencies and natural resource managers	−3	0	−1
26	MSPs with multiple stakeholder involvement complicate natural resource and landscape management; better leave it to experts	−3	0	2
27	MSPs are less useful than laws and regulations; we rather need law enforcement	−4	−3	0
28	MSPs only make sense if traditional authorities play important roles in them	0	1	0
29	MSPs do not make sense- eventually, all decisions are taken by government/experts	0	0	2
30	MSPs should be organized in a bottom-up manner	4	1	2
31	Participation of the private sector in MSPs is essential	0	2	−1
32	MSPs fail to consider multifunctional landscapes	−1	−1	−1
33	MSPs facilitate integrated land management and planning	1	1	2
34	MSPs are sustainable without external financing	−1	−4	0
35	MSPs are ineffective in delivering changes in practices and behavior regarding sustainable land uses	−1	1	1
36	MSPs are financed through projects	1	0	−2
37	MSPs are largely funded externally by the private sector, government, and donors	2	3	1
38	MSPs only work if communities have a say in decision-making regarding natural resources and landscape	2	2	0
39	MSPs are needed to negotiate trade-offs between different land users	1	4	1
40	MSPs do not effectively engage diverse stakeholders	−2	3	−3
41	MSPs do not improve collaborations among stakeholders	−2	−1	−1
42	MSPs only exist where there is a project	−1	−1	−1

In this discourse, MSPs are perceived as bottom-up institutions (#30) characterized by the participation of many players, including marginalized people (#2). This discourse recognizes the needs of local people (#18) and gives equal value to local and scientific knowledge (#8). More importantly, although adherents to this discourse recognize the private sector’s vital roles in decision-making and ensuring financial flows (#37), they do not consider the private sector fundamental to effectiveness, disagreeing with the statement, “Without the private sector, it is impossible to manage landscapes sustainably” (#24). According to this narrative, participants disagreed with the idea that MSPs are less useful than rules and regulations (#27) and are a cost burden and that decisions should be left to experts and government agencies (#25). This discourse emphasizes collective problem-solving, which thrives on democratic concepts of inclusion that allow equity to achieve common goals. The priorities of local people are meant to improve local livelihoods and entrench democracy in grassroots institutions.

Although some negative scores seem contradictory, they should be understood in the context, especially where stakeholders attempted to portray views of MSPs as enhancing grassroots democracy. According to the theory of rational decision-making under uncertainty, this behavior is justified because people rarely investigate new ways of viewing the world (Samuelson and Zeckhauser 1988). For example, adherents to this discourse perspective disagreed with the claim that “MSPs are not dominated by local chiefs” (#9). In practice, however, decisions made through local MSPs may need to be approved by the local chief before they can be implemented, a procedure that may cause some people to question the effectiveness of decisions made through MSPs, as in the statement: “MSPs do not make sense—eventually, all decisions are taken by government/experts” (#29). Similarly, while participants agreed that “MSPs are funded through projects” (#36), implying that without a project, the sustainability of MSPs is a concern, they disagreed that “MSPs only exist where there is a project” (#42).

#### Perspective 2: MSPs support market-based solutions committed to sustainable growth

This perspective was entirely dominated by respondents affiliated with state institutions, albeit in different government departments, and a private sector representative. This perspective emphasizes the importance of MSPs in promoting trade-off negotiations among various land uses (#39) and disagrees that MSPs hardly provide strategies that address land issues, e.g., conflicts over use (#20). In the study area, several interest groups are involved in negotiating trade-offs. These include stakeholder negotiations over conservation areas and development zones; over grazing and croplands between livestock farmers and agricultural producers; over forest over-use between charcoal entrepreneurs and renewable energy advocates; and over several other land uses. All these debates and negotiations occur in MSPs, such as Village Productivity Committees and sub-committees of the District Development Coordinating Committees. In some ways, the negotiations regard economic growth as a crucial concern for stakeholders to resolve in MSPs (#11). Participants in this discourse, for example, identified the necessity of MSPs recognizing and facilitating responsible trading in wood fuel by encouraging charcoal licensing (#4). However, the Kalomo landscape’s economic development trajectories are imbued with environmentally unsustainable production systems. In the post-Q-sort interviews, commercial tobacco and cotton farmers in the Kalomo District were highlighted as an example of those contributing to unsustainable land uses. This challenges ways in which conservation values and economic growth are balanced. Thus, proponents of this discourse argue that while “MSPs are useful for effective engagement with diverse stakeholders” (#1), they are less capable of resolving this complexity in their current forms since they do not effectively engage enough diversity of stakeholders (#40).

Another critical aspect of this narrative is the financing and sustainability of MSPs. Respondents disagreed with the statement that MSPs are sustainable without external funding (#34). Local MSPs undoubtedly have limited capacity to mobilize financing, and as such, they largely depend on the private sector or external donors to finance their activities (#37). For example, post-Q-sorting interviews identified a “powerful” seed company supporting the Chikanta Development Trust, composed of members of the council of elders and constituting a high-level village MSP. This point of view is telling in terms of private sector interests and influence in MSPs. As Oberlack et al. (2018) argue, globally, external interests such as the private sector and NGOs usually infuse their agenda, shaping discourses. In the study area, “seed companies and tobacco enterprises, under the guise of social responsibility, fund activities such as climate change-motivated tree-planting activities through local MSPs with the overt aim to increase their business footprint for profit” (male aged 54, Village head, interview 2021). This narrative is entrenched in the understanding that MSPs focus mainly on economic development (#11) while acknowledging that MSP resolutions contribute to improved land-use practices (#21).

#### Perspective 3: MSPs address power imbalances and identify policy gaps/needs

Respondents championing this discourse are persons affiliated with non-governmental organizations (Land Alliance, World Vision, and Zambia Community-Based Natural Resources Management Forum) and the private sector (a tobacco company undertaking farmer outreach in the study area).

Participants adhering to this discourse position MSPs as brokers that challenge the dominance of one group and propose to achieve greater power equity, empowerment, inclusion, and participation. The defining statement was that MSPs help address power imbalances (#7) and identify policy gaps (#13), as most policy impacts are at the local level. In a gendered society where women and youth continue to be marginalized, MSPs provide mechanisms through which their voices are heard, and their needs are prioritized (#18). More importantly, participants regarded MSPs as channels through which stakeholders equally engage each other (#12) to address land-use conflicts that emerge due to different power positions—women, youth, and vulnerable farmers often exploited by the “powerful”.

Those who adhered to this narrative, mainly civil society, strongly disagreed that MSPs should principally focus on discussing economic development issues (#11), such as commodity pricing, farmer production inputs, and timber concessions. As the post-sort interviews revealed, “farmers hardly control nor apprehend the price jargon beyond understanding farm-level production variables”. However, MSPs provide a constituency that considers redistribution of power. Primarily, the reason was that “local communities do not have the necessary capacity to engage with powerful private sector stakeholders and commercial farmers effectively” (post-sort interview with respondent M42, non-governmental organization participant, Kalomo, interview 2021). The emergence of civil society empowering local people in matters of access right to natural resources—especially marginalized women and youth, vulnerable farmers, and immigrants—creates a green radicalism discourse around MSPs that seeks equity and redressing power imbalances. Some NGOs that emphasize women’s empowerment and addressing gender injustice could be said to fit an ecofeminism sub-discourse in green radicalism.

### Consensus

**Fig. 5:**
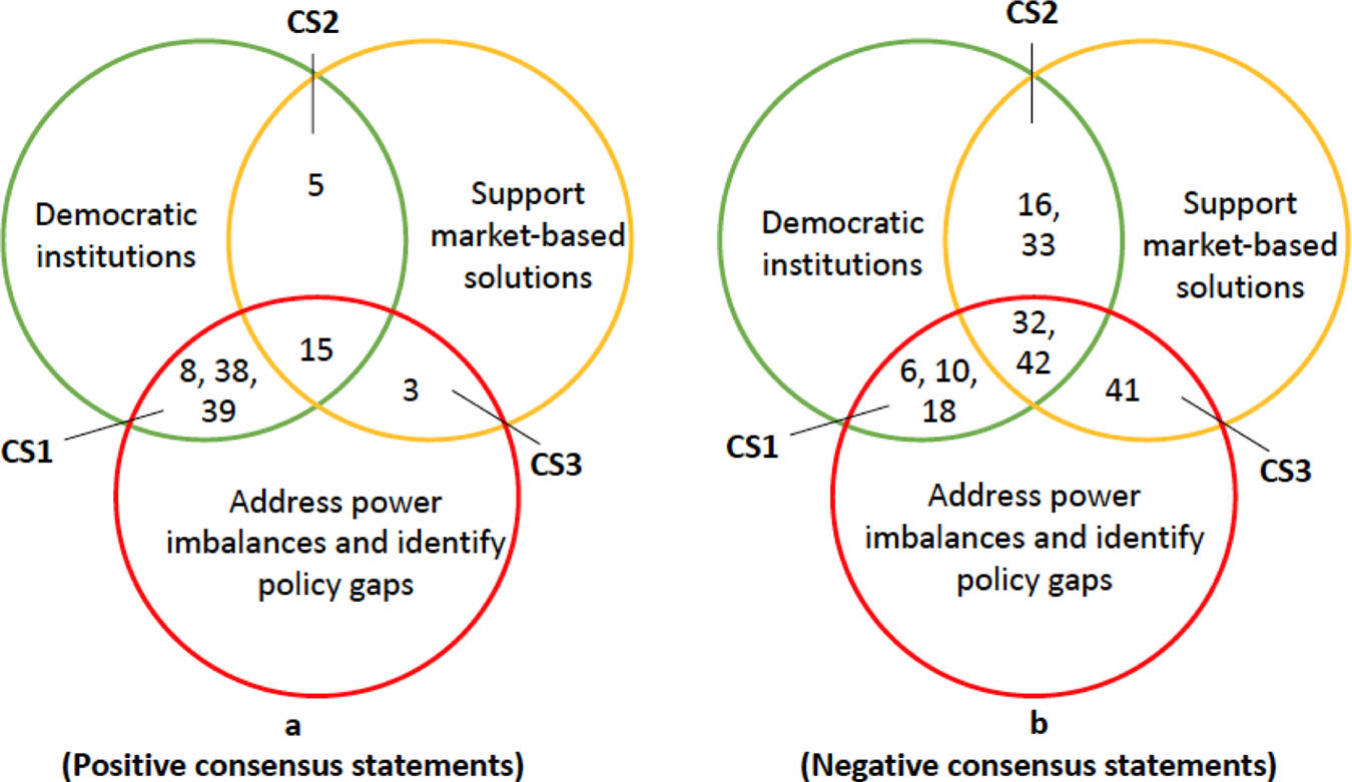
Positive (**a**) and negative (**b**) consensus statements across the three perspectives. *Key:* CS = Consensus statements. Note: the numbers shown in the Venn diagram represent the corresponding statement numbers in Table 3

There were some statement overlaps among the three discourse frames, i.e., between discourses about MSPs as democratic institutions for equitable and inclusive decision-making, forums that support market-based solutions committed to sustainable growth, address power imbalances, and identify policy gaps. Thus, the overlapping statements, referred to as consensus statements (CS in Fig. 5) (Zabala et al. 2018; Langston et al. 2019), provide insights into where ‘discourse coalitions’ emerge, might be explored, nurtured, and possibly leveraged. Figure 5 (a and b) shows agreed and disagreed CS among the three discourses. Detecting such overlaps helps identify common concern entry points (Sayer et al. 2013), although most CS did not directly advance much ‘purchasing power’ to explain common concern entry points. Nevertheless, I found three statements of particular importance with implications for ILAs.

In the context of a dual land-tenure system that includes both state and traditional governance systems, the collective thinking that MSPs facilitate the harmonization of policies and laws with traditional practices and regulations (#15) can be utilized to initiate a dialogue about common concerns to solve landscape challenges (ILA principle #2: common concern entry point).

Similarly, both democratic pragmatists and participants in a discourse that challenges power dynamics in MSPs agree that MSPs are institutions that give equal value to both local and scientific knowledge (#8). This consensus statement serves as a springboard for fruitful MSP deliberations that consider the equal worth of all forms of knowledge and knowledge holders and could thus support efforts at clarifying the rights of all stakeholders (ILA principle #2). This establishes the moral order of MSPs based on synergizing knowledge systems and their inclusion in negotiations, particularly in a setting where local priorities are not always recognized.

The democratic institution for equitable and inclusive decision-making and a discourse articulating power imbalances and identifying policy gaps disagreed that MSPs might be a source of conflict (#16). This viewpoint would help strengthen the common understanding that MSPs promote negotiated and transparent outcomes (ILA principle #6), which is necessary for the common concern entry point since it emphasizes the contribution of MSPs in trade-off negotiations.

## Discussion

This study examined different stakeholder perspectives on the role of MSPs in resolving social and environmental challenges in Kalomo District, Zambia. Over the last two decades, landscape governance scholars, policymakers, and practitioners have increasingly recognized MSPs as emergent governance frameworks that improve trade-off negotiations among various stakeholders. This paper untangled this narrative by relating field data to environmental discourses and scholarly work on landscape governance. Through the analysis of distinguishing statements, stakeholders articulated three perspectives.

The dissonance in some aspects of stakeholder perspectives on the role of MSPs in terms of policies, governance issues, funding, and laws and enforcement, suggests that landscape governance in Zambia is practiced in an environment marked by land-use tensions among stakeholders. Their differing expectations imply different conceptualizations of MSPs. This is unsurprising in a system of legal pluralism like Zambia, where problems are dealt with at various governance jurisdictions. The diversity of discourse framings observed in this study helps explain why landscape challenges have persisted at the district and sub-district levels despite numerous efforts to strengthen dialogue platforms. From the landscape governance theory point of view, the findings validate assumptions that ILA principles aim to foster collaborations in resolving issues (trade-offs) and reducing landscape tensions (Jeffrey 2009; Vermunt et al. 2020). Therefore, an adequate understanding of the roles of MSPs from various perspectives becomes important for ILA implementation.

The first perspective aligns with Dryzek’s (2013) description of democratic pragmatism as an environmental problem-solving sub-discourse, which conveys a way of thinking about and tackling environmental issues. Here, pragmatism refers to the flexibility that allows for the democratic inclusion of stakeholders’ broader value judgments in trying to reach decisions. The bottom-up type of MSP governance identified in this discourse is characterized by flexibility, which is a counter-model to technocratic and bureaucratic approaches promulgated in various policies in Zambia (e.g., environmental impact assessment processes) that are expert-focused and fit the administrative rationalism sub-discourse of environmental problem-solving. In the democratic pragmatism discourse, stakeholders highlight the bottom-up nature of MSPs, the necessity of private-sector participation, and the importance of prioritizing local needs and the demands of marginalized people while downplaying the usefulness of formal laws and regulations as the ultimate means to resolve landscape issues. In contrast, administrative rationalism—a “leave it to the experts” narrative that suggests leaving solutions to resource managers and bureaucrats (Dryzek 2013)—is a narrative that does not conform to democratic values of inclusiveness. A democracy-related discourse raises concerns about legitimacy, accountability, fairness, and representation (Olson 2011; Gleckman 2018). These concerns are important if MSPs are to deliver equitable decisions. In similar contexts, Ratner et al. (2022) established, from eight case studies across different landscapes, including Zambia, that most MSPs were established to foster inclusion and collaboration. In the absence of trust and other democratic norms in MSPs, negotiating trade-offs is difficult, and the governance paradigm mostly shifts to rely on dominant formal systems of rules and regulations, which may escalate conflicts. This discourse reflects the values of local MSPs as venues for the protection of local voices, such as in Village Productivity and Development committees. However, the discourse does not propose fundamentally altering power positions, distinguishing it from Dryzek’s green radicalism.

The second perspective appears to diverge from traditional thinking: bureaucrats or government experts merely rubber-stamp government policies and are more concerned with adhering to processes supporting economic trajectories rather than achieving socially desirable outcomes (Hoag and Hull 2017). Such assumptions about bureaucrats have their roots in classical sociology (Roy and Stone 1956) and continue to be echoed in contemporary studies on leadership until the new dawn of transformative leadership in the 1970s (Shaw 1992). My study reveals a narrative in which bureaucrats in Kalomo District regard MSPs as embracing economic development. The economic development focus in this MSP narrative is envisaged to support the sustainable supply of environmental goods and services, hinting at an economic rationalism stand, although it also somewhat bears some hallmarks of a sustainability discourse. However, the basic assumption of a sustainability discourse is that unrestrained material expansion cannot be reconciled with environmental sustainability, and as a result, economic development must be significantly reoriented to include sustainable actions. This implies that this combination of discourses is often untenable in practice (Awang et al. 2020). For example, the economic development agenda imposed on most countries in the global South is premised on extractive production systems based on the exploitation of raw materials that leave a trail of poverty rather than the development it envisions (Ansari et al. 2022). The proponents of the second perspective perceive MSPs as institutions that rationally promote economic growth and enable the private sector to contribute to sustainable development—a perspective also reflected in Upla et al. (2022), who assessed the potential role of the private sector in the value chain of commodities in the ILA context in Kalomo District. Such a perspective aligns with economic rationalism, a sub-discourse of problem-solving (Dryzek 2013), which is related to the worldview that environmental decisions should not hinder economic development (Goodpaster 1993). Inherent in this discourse, environmental solutions are embedded in capitalist free markets and risk placing MSPs to help commodify public goods through negotiating land titles, certification, payment for ecosystem services, and privatization (Gunderson 2017; Smessaert et al. 2020). This eventually excludes poor stakeholders. As was deduced from the statements aligning with the second perspective, government interference in environmental governance is limited to a certain extent perpetuating power inequalities.

Unsurprisingly, enhancing negotiation between the private sector stakeholders and poor farmers often is seen as a means of legitimizing power held by the powerful (McKeon 2017). Various natural resource management policies in Zambia theoretically support the co-existence of interests of landowners and the private sector to mutually maximize economic benefits from natural capital, e.g., joint forestry and community forest management. In practice, a perspective on MSPs that emphasizes the negotiation of economic incentives generally benefits private sectors over local people through forest concessions, public-private tobacco out-grower schemes, and farm trees. For this public-private relationship to be beneficial, well-functioning socially networked platforms are required (Djalante 2012), which is not the case in the study area as most MSPs are marred with weak stakeholder linkages (Reed et al. 2022). In this regard, bridging institutions are needed to mediate negotiation (Ros-Tonen et al. 2018).

The third discourse depicts a new order of an emerging green movement in environmental governance in Zambia comprising civil society stakeholders, comparable to what Martinez-Alier (2002) refers to as the “environmentalism of the poor”. These green movements have set their footprint in MSPs defined by various rights-based approaches worldwide, especially in the global South. The statements extracted through factor analysis revealed that CSOs were concerned with *power imbalances*, *policy gaps addressing equality*, and *solutions for addressing land concerns (conflicts over land use)*, reinforcing the discourse of green politics seen in many global forums. In this study, the third discourse identifies the role of MSPs in putting people’s rights at the center. This version of the analysis is consistent with the goals of the Zambia Community-Based Natural Resource Management Forum, a national MSP with a presence in local communities. The agenda of this consortium of CSOs, of which some were represented in this research, is to disrupt power inequalities and address rights and access to resources for locals. The discourse espoused by CSO-related participants centered on green politics that aim to influence collective awareness of environmentalism and effect political change in societal structures. The general philosophy of green radicalism focuses on social-political and power politics to deal with inequalities in decision-making. This discourse has the potential to be transformative in promoting a sustainable society based on a society aware of social justice issues and the need to build grassroots democracies through MSPs.

Different discourses typically conflict and can be difficult to harmonize. One of the most interesting outcomes of the three discourse frames is the stakeholders’ tendency to cluster around common narratives, a trend one would describe as “like-mindedness” (based on some general patterns of selecting statements but highly dependent on the topic of interest) (di Gregorio et al. 2019). This like-mindedness holds the potential to serve as a starting point for improving engagement in decision-making, particularly in the context of environmental governance, where varied perspectives are common. Given the dual land tenure system that oscillates between the customary and state governance systems, the consensus among all the discourses concerning the need for MSPs to harmonize policies and laws with traditional customs and regulations demonstrates a collective desire to solve a common problem, a feature that resonates with the ILA principle related to the common concern entry point. This is especially significant given the involvement of government institutions, traditional leaders, the commercial sector, local-level groups, and civil society. It is also worth mentioning that ILAs attempt to clarify stakeholders’ responsibilities and rights by facilitating the identification of possible commitments from diverse stakeholders (Sayer et al. 2013). In this sense, knowing and identifying knowledge holders (scientific or local) helps facilitate an inclusive and transparent process.

In contrast to other research that claims ILAs perpetuate neoliberal conservation and reinforce power imbalances (Clay 2016), respondents in this study seemed to have found MSPs, which are key to ILAs, to assist in resolving conflicts and promoting equitable negotiations. This consensus among different perspectives is an example of how new perspectives that stimulate dialogue are revealed, creating stakeholder networks within and across participants sharing similar expectations. In the policy context, uncovering consensus statements scrutinized and later distilled from various discourse framings is essential to focus the dialogue toward resolving challenges in a “win-more-lose-less” situation (Langston et al. 2019).

As this Q-study showed, participants demonstrated that MSPs could play significant roles in addressing locally embedded problems, but this is contingent on the issues that need to be addressed and the composition of stakeholders. This is important for the development of the ILA theory (Boedhihartono et al. 2018). This study helps to contextualize how and why different perspectives on MSP governance emerge. In doing so, it contributes to the literature on landscape governance by demonstrating why it is critical to recognize stakeholder differences and consensus in framing MSPs. This helps start a new research conversation emphasizing the need to consider stakeholder discourses distilled from the local experiences within collaborative approaches (Toomey et al. 2017).

However, further research is needed to address some limitations of this study. Although the analysis may have a broader application to other similar tropical contexts, the focus of this study was exclusively on a case study, and the findings should be interpreted in the specific setting of this work. Second, considering that Q-methodology is a semi-quantitative technique that depends on a small number of experts, it can potentially miss other points of view, particularly at the community level and those of illiterate stakeholders who cannot participate in a Q-exercise. Third, further research is recommended to explore further how different perspectives are linked to specific stakeholder positions. Fourth, the Q-methodology may not be suitable for application and policy development at the macro level (national and above). It is still necessary to conduct additional research at the macro level to understand the implications of the findings for long-term landscape sustainability. Fifth, the financial implications of MSPs must also be explored further, as this was not sufficiently addressed in this study. This can include additional perspectives from donors, who, while not often present in landscapes, have an impact on MSP activities.

## Conclusion

Using the Q-methodology, this study uncovered three stakeholder perspectives regarding the role of MSPs in landscape governance, providing insight into why stakeholders in the Kalomo landscape in Zambia struggle to identify and address common concerns. Because perspectives shown in the discourses are so varied, reconciling opposing points of view is complicated. In this paper, I deduce that the efficiency of MSPs in delivering outcomes in terms of identifying and addressing common concerns and negotiated solutions cannot be detached from how stakeholders perceive these MSPs. Nevertheless, the divergence in perceptions also allows for identifying common markers as entry points for dialogue.

The three perspectives are conceptually diverse. The first perspective is that MSPs should be presented as inclusive institutions promoting dialogue. This contrasts with the second discourse in which, despite acknowledging the diversity of stakeholders in MSPs, the roles of the government and private sectors are more prominently outlined. As such, MSPs should focus on mediating conflicts among various land users. This viewpoint, like the first one, emphasizes the presence of marginalized stakeholders and their needs. However, they differ from the third discourse, anchored in a green radicalism discourse. Unlike the first two discourses, the third one recognizes power imbalances and how MSPs can act as brokers by guaranteeing equal power distribution in decision-making. Despite these differences, all perspectives identified MSPs as having the potential to harmonize policies in a dual governance system. Moreover, they imply that MSPs foster more equitable dialogue between stakeholders toward a transformative change in landscape governance, thereby confirming that MSPs are key to implementing ILAs.

### Supplementary information

Supplementary Information

## Footnotes

1 A theory of change is an agreed course of action within a project or initiative toward a commonly desired future, often used as a basis for evaluation (Sayer et al. 2017; Reed et al. 2022).

2 The Collaborating to Operationalize Landscape Approaches for Nature, Development and Sustainability (COLANDS) initiative is led by the International Centre for Forestry Research (CIFOR) with the University of British Columbia, University of Amsterdam, and the French agricultural research and international cooperation organisation working for the sustainable development of tropical and Mediterranean regions (CIRAD). With several local partners in the countries of implementation—Ghana, Zambia, and Indonesia—the COLANDS initiative seeks to initiate, analyze and evaluate the implementation of ILAs. For more information, see https://www.cifor-icraf.org/colands/.

## References

[CR1] Ansari MA, Villanthenkodath MA, Akram V, Rath BN (2022) The nexus between ecological footprint, economic growth, and energy poverty in sub-Saharan Africa: a technological threshold approach. Environ Dev Sustain 1–28. 10.1007/s10668-022-02377-5

[CR2] Armatas C, Venn T, Watson A (2017). Understanding social-ecological vulnerability with Q-methodology: a case study of water-based ecosystem services in Wyoming, USA. Sustain Sci.

[CR3] Awang AH, Haron M, Zainuddin Rela I, Saad S (2020). Formation of civil servants’ creativity through transformative leadership. J Manag Dev.

[CR4] Barletti. JPS, Larson M A (2019) The role of multi-stakeholder forums in subnational jurisdictions: Methods training manual and tools for in-depth research. The role of multi-stakeholder forums in subnational jurisdictions: Methods training manual and tools for in-depth research. 10.17528/cifor/007149

[CR5] Barry J, Proops J (1999). Seeking sustainability discourses with Q methodology. Ecol Econ.

[CR6] Bateson G, Gregory B (1972). A theory of play and phantasy. Steps to an ecology of the mind: Collected essays in Anthropology, Psychiatry, Evolution and Epistemology.

[CR7] Biersteker E, van Marrewijk A, Koppenjan J (2022). Identifying Subjective Perspectives on Managing Underground Risks at Schiphol Airport. Proj Manag J.

[CR8] Boedhihartono AK, Bongers F, Boot RGA (2018). Conservation Science and Practice Must Engage With the Realities of Complex Tropical Landscapes. Trop Conserv Sci.

[CR9] Brown SR (1993). A Primer on Q Methodology. Operant Subjectivity.

[CR10] Brown TA (2015) Confirmatory Factor Analysis, 2nd edn. The Guilford Press, New York

[CR11] Buckwell A, Fleming C, Muurmans M, Smart JCR, Ware D, Mackey B (2020). Revealing the dominant discourses of stakeholders towards natural resource management in Port Resolution, Vanuatu, using Q-method. Ecol Econ.

[CR12] Byamugisha FF (Ed.) (2014) Agricultural land redistribution and land administration in sub-Saharan Africa: case studies of recent reforms. World Bank Publications, Washington DC

[CR13] Carpentier N, Doudaki V, Rozsypal Pajerová A (2021). Conflicting and entangled human–nature relationships: A discursive-material analysis of the documentary film Kiruna - A Brand New World. People Nat.

[CR14] Chilombo A (2021). Multilevel governance of large-scale land acquisitions: a case study of the institutional politics of scale of the farm block program in Zambia. Land Use Policy.

[CR15] Chong D, Druckman JN (2007). Framing theory. Annu Rev Political Sci.

[CR16] Clay N (2016). Producing hybrid forests in the Congo Basin: a political ecology of the landscape approach to conservation. Geoforum.

[CR17] Dale VH, Kline KL, Parish ES, Eichler SE (2019). Engaging stakeholders to assess landscape sustainability. Landsc Ecol.

[CR18] Dentoni D, Bitzer V, Schouten G (2018). Harnessing Wicked Problems in Multi-stakeholder Partnerships. J Bus Ethics.

[CR19] Djalante R (2012). Adaptive governance and resilience: the role of multi-stakeholder platforms in disaster risk reduction. Nat Hazards Earth Syst Sci.

[CR20] Dryzek J (2013). The Politics of the Earth: Environmental Discourses, Third.

[CR21] Dryzek JS, Niemeyer S (2008). Discursive representation. Am Political Sci Rev.

[CR22] Dube K, Nhamo G (2019). Climate change and potential impacts on tourism: evidence from the Zimbabwean side of the Victoria Falls. Environ Dev Sustain.

[CR23] Faysse N (2006). Troubles on the way: an analysis of the challenges faced by multi-stakeholder platforms. Nat Resour Forum.

[CR24] Fowler A, Biekart K (2017). Multi-Stakeholder Initiatives for Sustainable Development Goals: the Importance of Interlocutors. Public Adm Dev.

[CR25] Geels FW, McMeekin A, Mylan J, Southerton D (2015). A critical appraisal of Sustainable Consumption and Production research: the reformist, revolutionary and reconfiguration positions. Glob Environ Change.

[CR26] Gleckman H (2018). Multistakeholder Governance and Democracy.

[CR27] Goodpaster G (1993). Rational Decision-Making in Problem-Solving Negotiation: Compromise, Interest-Valuation, and Cognitive Error. Ohio State J Disput Resolut.

[CR28] di Gregorio M, Fatorelli L, Paavola J (2019). Multi-level governance and power in climate change policy networks. Glob Environ Change.

[CR29] Gruber J (2011). Perspectives of effective and sustainable community-based natural resource management: an application of Q methodology to forest projects. Conserv Soc.

[CR30] Gunderson R (2017) Commodification of nature. In: Richardson D, Castree N, Goodchild MF, Kobayashi A, Liu W, Marston RA (eds) International Encyclopedia of Geography: People, the Earth, Environment and Technology, Wiley, Hoboken NJ, p. 1–20

[CR31] Hermans F, Sartas M, van Schagen B (2017). Social network analysis of multi-stakeholder platforms in agricultural research for development: Opportunities and constraints for innovation and scaling. PLoS ONE.

[CR32] Hermans F, Kok K, Beers PJ, Veldkamp T (2012). Assessing Sustainability Perspectives in Rural Innovation Projects Using Q-Methodology. Socio Ruralis.

[CR33] Hjortskov M (2019). Citizen Expectations and Satisfaction Over Time: Findings From a Large Sample Panel Survey of Public School Parents in Denmark. Am Rev Public Adm.

[CR34] Hoag C, Hull M (2017) A Review of the Anthropological Literature on the Civil Service. Policy Research Working Paper No. 8081, World Bank, Washington, DC

[CR35] Ihemezie EJ, Stringer LC, Dallimer M (2022) Understanding the diversity of values underpinning forest conservation. Biol Conserv 274. 10.1016/j.biocon.2022.109734

[CR36] Jeffrey S (2009) Reconciling Conservation and Development: Are Landscapes the Answer? Biotropica 41:649–652. 10.1111/j.1744-7429.2009.00575.x

[CR37] Kampen JK, Tamás P (2014). Overly ambitious: contributions and current status of Q methodology. Qual Quant.

[CR38] Kusters K, de Graaf M, Buck L (2020). Inclusive landscape governance for sustainable development: Assessment methodology and lessons for civil society organizations. Land (Basel).

[CR39] Langston JD, McIntyre R, Falconer K (2019). Discourses mapped by Q-method show governance constraints motivate landscape approaches in Indonesia. PLoS ONE.

[CR40] Larson AM, Sarmiento Barletti JP, Heise Vigil N (2022). A place at the table is not enough: Accountability for Indigenous Peoples and local communities in multi-stakeholder platforms. World Dev.

[CR41] Mahlalela LS, Jourdain D, Mungatana ED, Lundhede TH (2022). Diverse stakeholder perspectives and ecosystem services ranking: Application of the Q-methodology to Hawane Dam and Nature Reserve in Eswatini. Ecol Econ.

[CR42] Mani-Peres C, Xavier LY, Santos CR, Turra A (2016). Stakeholders perceptions of local environmental changes as a tool for impact assessment in coastal zones. Ocean Coast Manag.

[CR43] Martinez-Alier J (2002). The Environmentalism of the Poor.

[CR44] McKeon N (2017). Are Equity and Sustainability a Likely Outcome When Foxes and Chickens Share the Same Coop? Critiquing the Concept of Multistakeholder Governance of Food Security. Globalizations.

[CR45] Metzger J, Soneryd L, Linke S (2017). The legitimization of concern: A flexible framework for investigating the enactment of stakeholders in environmental planning and governance processes. Environ Plan A.

[CR46] Minkman E, van der Sanden M, Rutten M (2017). Practitioners’ viewpoints on citizen science in water management: A case study in Dutch regional water resource management. Hydrol Earth Syst Sci.

[CR47] Moombe KB, Siangulube FS, Mwaanga BM, Reed J, Ros-Tonen MAF, Sunderland T (2020). Understanding landscape dynamics A case study from Kalomo District. Operationalizing integrated landscape approaches in the tropics.

[CR48] Muchimba D (2022) An investigation into land conflicts in Kalomo district-Zambia: an analysis of the process of land acquisition (Doctoral dissertation). University of Zambia, Lusaka

[CR49] Newig J, Günther D, Pahl-Wostl C (2010). Social Network Analysis in Natural Resource Governance Synapses in the Network: Learning in Governance Networks in the Context of Environmental Management. Ecol Soc.

[CR50] Oberlack C, Boillat S, Brönnimann S, et al (2018) Polycentric governance in telecoupled resource systems. Ecol Soc 23: 10.5751/ES-09902-230116

[CR51] Olson K (2011) Deliberative democracy. In: Fultina B (ed) Jürgen Habermas: Key Concepts. Acumen Publishing Limited, Stocksfield UK, p. 140–155

[CR52] Ratner BD, Rivera A, Fiorenza A (2019). Engaging government for policy influence through multi-stakeholder platforms.

[CR53] Ratner BD, Larson AM, Sarmiento Barletti JP (2022). Multistakeholder platforms for natural resource governance: lessons from eight landscape-level cases. Ecol Soc.

[CR54] Ray DK, West PC, Clark M (2019). Climate change has likely already affected global food production. PLoS ONE.

[CR55] Reed J, Oldekop J, Barlow J, Carmenta R, Geldmann J (2020). The extent and distribution of joint conservation-development funding in the tropics. One Earth.

[CR56] Reed J, Chervier C, Borah JR, et al (2022) Co-producing theory of change to operationalize integrated landscape approaches. Sustain Sci. 10.1007/s11625-022-01190-3 10.1007/s11625-022-01190-3 PMC9465133 36119558

[CR57] Ros-Tonen MAF, Reed J, Sunderland T (2018). From Synergy to Complexity: The Trend Toward Integrated Value Chain and Landscape Governance. Environ Manag.

[CR58] Roy FG, Stone RC (1956) Service and Procedures in Bureaucracy, NED-New. University of Minnesota Press, Minneapolis, Minnesota

[CR59] Samuelson W, Zeckhauser R (1988). Status quo bias in decision making. J Risk Uncertain.

[CR60] Sarmiento Barletti JP, Larson AM, Heise Vigil N (2022). Understanding Difference to Build Bridges among Stakeholders: Perceptions of Participation in Four Multi-stakeholder Forums in the Peruvian Amazon. J Dev Stud.

[CR61] Sarmiento-Barletti JP, Larson AM (2019). The role of multi-stakeholder forums in subnational jurisdictions.

[CR62] Sartas M, Schut M, Hermans F et al. (2018) Effects of multi-stakeholder platforms on multi-stakeholder innovation networks: Implications for research for development interventions targeting innovations at scale. PLoS ONE 13: 10.1371/journal.pone.0197993 10.1371/journal.pone.0197993 PMC5988278 29870559

[CR63] Sayer J, Sunderland T, Ghazoul J (2013). Ten principles for a landscape approach to reconciling agriculture, conservation, and other competing land uses. Proc Natl Acad Sci.

[CR64] Sayer JAJA, Margules C, Boedhihartono AK (2017). Measuring the effectiveness of landscape approaches to conservation and development. Sustain Sci.

[CR65] Shaw CKY (1992). Hegel’s Theory of Modern Bureaucracy. Am Political Sci Rev.

[CR66] Siangulube FS, Ros-Tonen MAF, Reed J, Djoudi H, Gumbo D, Sunderland T (2023). Navigating power imbalances in landscape governance: a network and influence analysis in Southern Zambia. Reg Environ Change.

[CR67] Smessaert J, Missemer A, Levrel H (2020). The commodification of nature, a review in social sciences. Ecol Econ.

[CR68] Smith R (2005). Land tenure and farm performance in Zambia’s Southern Province.

[CR69] Stephenson W (1935) Correlating Persons instead of tests. J Pers 4. 10.1111/j.1467-6494.1935.tb02022.x

[CR70] Stone LS, Stone MT, Mogomotsi PK, Mogomotsi GE (Eds.) (2022) Protected areas and tourism in Southern Africa: conservation goals and community livelihoods. Routledge, Abingdon/New York

[CR71] Summak M, Kalman M (2020). A Q-methodological analysis of school principals’ decision-making strategies during the change process at schools. Cent Educ Policy Stud J.

[CR72] Tannen D, Tanteen D (1993). Introduction. Framing in discourses.

[CR73] Thapa B (2012). Why did they not visit? Examining structural constraints to visit Kafue National Park, Zambia. J Ecotourism.

[CR74] Toomey AH, Knight AT, Barlow J (2017). Navigating the Space between Research and Implementation in Conservation. Conserv Lett.

[CR75] Upla P, Reed J, Moombe KB, et al (2022) Assessing the Potential for Private Sector Engagement in Integrated Landscape Approaches: Insights from Value-Chain Analyses in Southern Zambia. Land (Basel) 11. 10.3390/land11091549

[CR76] van Ewijk E, Ros-Tonen MAF (2021). The fruits of knowledge co-creation in agriculture and food-related multi-stakeholder platforms in Sub-Saharan Africa – a systematic literature review. Agric Syst.

[CR77] van Loenen B (1999) Land tenure in Zambia. University of Maine, Orono. https://www.researchgate.net/publication/242672704_Land_tenure_in_Zambia

[CR78] Velde KV, Huge J, Friess DA, Koedam N, Dahdouh-Guebas F (2019). Stakeholder discourses on urban mangrove conservation and management. Ocean Coast Manag.

[CR79] Vermunt DA, Verweij PA, Verburg RW (2020) What Hampers Implementation of Integrated Landscape Approaches in Rural Landscapes? Current Landscape Ecology Reports. 10.1007/s40823-020-00057-6

[CR80] Warner JF (2006). More sustainable participation? Multi-Stakeholder Platforms for integrated catchment management. Int J Water Resour Dev.

[CR81] Watts S (2015). Develop a Q methodological study. Educ Prim Care.

[CR82] Watts S, Stenner P (2012). Doing Q methodological research: theory, method and interpretation.

[CR83] Whyte AVT (1977) Guidelines for field studies in environmental perception. In: MAB Technical Notes 5. UNESCO, Paris

[CR84] Zabala A, Pascual U (2016). Bootstrapping Q Methodology to Improve the Understanding of Human Perspectives. PLoS ONE.

[CR85] Zabala A, Sandbrook C, Mukherjee N (2018). When and how to use Q methodology to understand perspectives in conservation research. Conserv Biol.

[CR86] Zanella MA, Goetz A, Rist S et al. (2018) Deliberation in multi-stakeholder participation: a heuristic framework applied to the Committee on World Food Security. Sustainability (Switzerland) 10: 10.3390/su10020428

