# Supplementary material for: The Role of Multistakeholder Platforms in Environmental Governance: Analyzing Stakeholder Perceptions in Kalomo District, Zambia, Using Q-Method
Source: Environ Manage. 2023 Mar 20;74(1):13–30. doi: 10.1007/s00267-023-01806-z (PMC11208185; doi:10.1007/s00267-023-01806-z)
Supplement: Supplementary file 1 — Supplementary Information [file 267_2023_1806_MOESM1_ESM.docx]

**Supplementary Material**

# Appendix 1: Orientational strands of MSPs in the Kalomo District based on participants interviewed

| Orientation | Key roles | Whom are they targeting | Who has the principal capacity to act | Examples |
| --- | --- | --- | --- | --- |
| Development-focused interventions | Negotiate long-term social development and amenities in the landscapes. | Local policymakers (councilors) and Development partners (local councils) | Local councils | District development coordinating committees/ constituency development committees |
| Conflict management | Resolution of conflicts and sustainability of land-use practices | Land users, traditional leaders, and development planners | Local leaders | Village-level MSPs |
| Conservation focused | Long-term sustainability of social-ecological systems | Conservation NGOs, local resource users | Resource users | ZCBNRMF^a^ |
| Research for Development and Conservation (R4D-C) | Provision of research-based solutions and communication of information for policy development | Researchers, scientists, government departments, local councils, and local communities | All | District Consultative group |
| Public-private partnership | Ensure continuity of financial flows from private to public initiatives | Government and private sector | All | Kalomo District Chamber of Commerce |
| Rights-based/focused | Empowerment of local people at the village level and rights claims in decision-making in land matters. | Advocacy NGOs and marginalized groups | Empowered marginalized groups | Village-level MSPs speared by Women for Change/ women groups/ cooperatives |

*Source*: Inventory made by the author during fieldwork in Kalomo District in 2020.

^a^ Zambia Community-Based Natural Resource Management Forum

# Appendix 2: Example of a Q-sort grid used in the study

| **-4** | **-3** | **-2** | **-1** | **0** | **+1** | **+2** | **+3** | **+4** |
| --- | --- | --- | --- | --- | --- | --- | --- | --- |
|  |  |  |  |  |  |  |  |  |
|  |  |  |  |  |  |  |  |  |
|  |  |  |  |  |  |  |  |  |
|  |  |  |  |  |  |  |  |  |
|  |  |  |  |  |  |  |  |  |
|  |  |  |  |  |  |  |  |  |
|  |  |  |  |  |  |  |  |  |
|  |  |  |  |  |  |  |  |  |
|  |  |  |  |  |  |  |  |  |
|  |  |  |  |  |  |  |  |  |

# Appendix 3: Factor matrix with defining Q-sorts

| No. | Participants’ affiliation^b^ | **MSPs as democratic institutions** | Flagged^a^ | MSPs support market-based solutions | Flagged | MSPs address power imbalance and identify policy gaps | Flagged |
| --- | --- | --- | --- | --- | --- | --- | --- |
| 1 | State actor 1 | 0.134 |  | 0.8956 | * | -0.0678 |  |
| 2 | State actor 2 | -0.0579 |  | 0.6562 | * | 0.0501 |  |
| 3 | State actor 3 | 0.1497 |  | 0.9109 | * | -0.0478 |  |
| 4 | State actor 4 | 0.073 |  | 0.7938 | * | 0.2304 |  |
| 5 | State actor 5 | 0.1976 |  | 0.7727 | * | -0.0082 |  |
| 6 | State actor 6 | 0.161 |  | 0.8924 | * | 0.0247 |  |
| 7 | State actor 7 | 0.2251 |  | 0.898 | * | -0.043 |  |
| 17 | State actor 8 | 0.6078 | * | -0.1526 |  | 0.0015 |  |
| 9 | CSO 1 | 0.1724 |  | -0.0377 |  | 0.9526 | * |
| 10 | CSO 2 | 0.1223 |  | -0.0563 |  | 0.9476 | * |
| 11 | CSO 3 | 0.106 |  | 0.1167 |  | 0.8673 | * |
| 18 | CSO 4/ CBO 1 | 0.9659 | * | 0.1548 |  | 0.0813 |  |
| 19 | CSO 5/ CBO 2 | 0.9539 | * | 0.1919 |  | 0.0958 |  |
| 23 | CSO 6/ CBO 3 | 0.8982 | * | 0.2488 |  | 0.0496 |  |
| 12 | Local leader 1 | 0.9539 | * | 0.1919 |  | 0.0958 |  |
| 13 | Local leader 2 | 0.875 | * | 0.288 |  | 0.0308 |  |
| 14 | Local leader 3 | 0.8255 | * | 0.153 |  | -0.1676 |  |
| 24 | Local leader 4 | 0.7968 | * | -0.0806 |  | -0.0314 |  |
| 8 | Private sector 1 | 0.0377 |  | 0.8134 | * | -0.0364 |  |
| 15 | Private sector 2 | 0.8603 | * | 0.1304 |  | -0.1335 |  |
| 16 | Private sector 3 | 0.3187 |  | -0.0295 |  | -0.6651 | * |
| 20 | Researcher 1 | 0.9539 | * | 0.1919 |  | 0.0958 |  |
| 21 | Researcher 2 | 0.9429 | * | 0.2092 |  | 0.1099 |  |
| 22 | Development organization | 0.9471 | * | 0.1935 |  | 0.1249 |  |
| 25 | Village member | 0.7968 | * | -0.0806 |  | -0.0314 |  |
| % explained variance | | 42 |  | 24 |  | 13 |  |

^b^ Names of respondents have been withheld to guarantee anonymity.

# Appendix 4: Q-sort with Z-scores and ranks in all the factors

| No. | Statement | MSPs as democratic institutions | | MSPs support market-based solutions | | MSPs address power imbalance and identify policy gaps | |
| --- | --- | --- | --- | --- | --- | --- | --- |
|  |  | Z-score | Rank | Z-score | Rank | Z-score | Rank |
| 1 | MSPs are useful for effective engagement with diverse actors | 0.19 | 17 | 0.62 | 14 | -1.94 | 42 |
| 2 | MSPs create an environment for marginalized people to participate | 1.5 | 3 | -1.1 | 37 | 0.44 | 14 |
| 3 | MSPs don’t engage in national policy matters | 0.14 | 18 | -0.62 | 32 | 0.03 | 23 |
| 4 | MSPs recognize the importance of charcoal licensing | 0.52 | 13 | 0.75 | 11 | -0.55 | 31 |
| 5 | MSPs engage national policymakers | 1.09 | 8 | 1.08 | 13 | 0.3 | 15 |
| 6 | MSPs should always have legal status | -0.52 | 27 | -1.65 | 33 | -0.64 | 39 |
| 7 | MSPs address power imbalances | 1.2 | 5 | 0.04 | 22 | 1.99 | 1 |
| 8 | MSPs give equal value to local and scientific knowledge | 0.43 | 15 | -1 | 36 | 0.52 | 13 |
| 9 | MSPs are not dominated by local chiefs | -0.57 | 28 | 1.17 | 7 | 0.09 | 18 |
| 10 | MSPs focus mainly on environmental issues | -0.92 | 34 | 0.05 | 21 | -1.12 | 35 |
| 11 | MSPs focus mainly on economic development | 0.57 | 12 | 1.23 | 4 | -1.67 | 40 |
| 12 | MSPs allow equitable participation | -1.14 | 36 | 0 | 23 | 1.31 | 5 |
| 13 | MSPs help identifies policy gaps | -1.7 | 39 | -0.81 | 35 | 1.72 | 2 |
| 14 | MSPs enable private sector participation and investment | 1.06 | 9 | -0.61 | 31 | -1.12 | 36 |
| 15 | MSPs facilitate the harmonization of policies and laws | 1.83 | 19 | 1.74 | 39 | 1.96 | 34 |
| 16 | MSPs can be a source of conflicts | -1.14 | 37 | -1.08 | 34 | -0.06 | 24 |
| 17 | MSPs do not help increase communication across sectors | 0 | 20 | -1.84 | 40 | 0 | 21 |
| 18 | MSPs recognize and prioritize local needs | 1.41 | 4 | 1.2 | 6 | 1.64 | 3 |
| 19 | MSPs hinder the adoption of best practices | 0 | 21 | -0.3 | 26 | 0 | 22 |
| 20 | MSPs hardly provide strategies that address land issues, e.g., conflict over use | 0 | 22 | -1.23 | 38 | 1.64 | 4 |
| 21 | MSPs resolutions contribute to improved land use practices activities | 0.61 | 11 | 0.9 | 9 | -0.11 | 26 |
| 22 | MSPs foster sharing of land use practices/ experiences | 1.61 | 23 | -0.03 | 5 | -0.46 | 29 |
| 23 | Only the private sector has the innovative capacity to manage landscape sustainability | 0 | 24 | -0.59 | 30 | 0.22 | 16 |
| 24 | Without the private sector, it is impossible to manage landscapes sustainably | -0.57 | 29 | -0.25 | 25 | 1.12 | 6 |
| 25 | MSPs only cost money. Better leave it to government agencies and natural resource managers | -1.7 | 40 | 0.06 | 20 | -0.54 | 30 |
| 26 | MSPs with multiple stakeholder involvement complicate natural resource and landscape management; better leave it to experts | -1.83 | 41 | 0.24 | 17 | -1.18 | 38 |
| 27 | MSPs are less useful than laws and regulations; we rather need law enforcement | -1.91 | 42 | -1.84 | 41 | 0.11 | 17 |
| 28 | MSPs only make sense if traditional authorities play important roles in them | 0 | 25 | 0.5 | 15 | -0.08 | 25 |
| 29 | MSPs do not make sense- eventually, all decisions are taken by government/experts | -0.05 | 26 | 0.18 | 18 | 1.12 | 7 |
| 30 | MSPs should be organized in a bottom-up manner | 2.16 | 1 | 0.76 | 10 | 1.06 | 8 |
| 31 | Participation of the private sector in MSPs is essential | 0 | 2 | 1.22 | 24 | -0.17 | 27 |
| 32 | MSPs fail to consider multifunctional landscapes | -0.57 | 30 | -0.51 | 29 | -0.74 | 33 |
| 33 | MSPs facilitate integrated land management and planning | -0.52 | 14 | -0.69 | 12 | 1.01 | 9 |
| 34 | MSPs are sustainable without external financing | -0.57 | 31 | -2.1 | 42 | 0.03 | 20 |
| 35 | MSPs are ineffective in delivering changes in practices and behavior regarding sustainable land uses | -0.57 | 32 | 0.45 | 16 | 0.66 | 11 |
| 36 | MSPs are financed through projects | 0.43 | 16 | 0.06 | 19 | -1.15 | 37 |
| 37 | MSPs are largely funded externally by the private sector, government, and donors | 1.12 | 7 | 0.8 | 2 | 1.6 | 12 |
| 38 | MSPs only work if communities have a say in decision-making regarding natural resources and landscape | 1.17 | 6 | 0.03 | 8 | 1.18 | 19 |
| 39 | MSPs are needed to negotiate trade-offs between different land users | 0.71 | 10 | 2.18 | 1 | 0.85 | 10 |
| 40 | MSPs do not effectively engage diverse actors | -1.14 | 38 | 1.38 | 3 | -1.69 | 41 |
| 41 | MSPs do not improve collaborations among actors | -1.11 | 35 | -0.44 | 27 | -0.89 | 32 |
| 42 | MSPs only exist where there is a project | -0.58 | 33 | -0.45 | 28 | -0.35 | 28 |

# Appendix 5: Legend for Z-scores in all the factors shown in Figure 3

|  | Statement Number | MSPs as democratic institutions | MSPs support market-based solutions | MSPs address power imbalances and identify policy gaps |
| --- | --- | --- | --- | --- |
| 13 | MSPs help identify policy gaps | -1,7 | -0,81 | 1,72 |
| 11 | MSPs focus mainly on economic development | 0,57 | 1,23 | -1,67 |
| 7 | MSPs address power imbalances | 1,2 | 0,04 | 1,99 |
| 26 | MSPs with multiple stakeholder involvement complicate natural resource and landscape management; better leave it to experts | -1,83 | 0,24 | -1,18 |
| 2 | MSPs create an environment for marginalized people to participate | 1,5 | -1,1 | 0,44 |
| 40 | MSPs do not effectively engage diverse actors | -1,14 | 1,38 | -1,69 |
| 1 | MSPs are useful for effective engagement with diverse actors | 0,19 | 0,62 | -1,94 |
| 9 | MSPs are not dominated by local chiefs | -0,57 | 1,17 | 0,09 |
| 12 | MSPs allow equitable participation | -1,14 | 0 | 1,31 |
| 25 | MSPs only cost money. Better leave it to government agencies and natural resource managers | -1,7 | 0,06 | -0,54 |
| 14 | MSPs enable private sector participation and investment | 1,06 | -0,61 | -1,12 |
| 20 | MSPs hardly provide strategies that address land issues, e.g., conflicts over use | 0 | -1,23 | 1,64 |
| 34 | MSPs are sustainable without external financing | -0,57 | -2,1 | 0,03 |
| 22 | MSPs foster sharing of land-use practices/experiences | 1,61 | -0,03 | -0,46 |
| 36 | MSPs are financed through projects | 0,43 | 0,06 | -1,15 |
| 24 | Without the private sector, it is impossible to manage landscapes sustainably | -0,57 | -0,25 | 1,12 |
| 29 | MSPs do not make sense- eventually, all decisions are taken by government/experts | -0,05 | 0,18 | 1,12 |
| 4 | MSPs recognize the importance of charcoal licensing | 0,52 | 0,75 | -0,55 |
| 21 | MSPs resolutions contribute to improved land-use practices | 0,61 | 0,9 | -0,11 |
| 27 | MSPs are less useful than laws and regulations; we rather need law enforcement | -1,91 | -1,84 | 0,11 |
| 6 | MSPs should always have legal status | -0,52 | -1,65 | -0,64 |
| 30 | MSPs should be organized in a bottom-up manner | 2,16 | 0,76 | 1,06 |
| 31 | Participation of the private sector in MSPs is essential | 0 | 1,22 | -0,17 |
| 33 | MSPs facilitate integrated land management and planning | -0,52 | -0,69 | 1,01 |
| 35 | MSPs are ineffective in delivering changes in practices and behavior regarding sustainable land uses | -0,57 | 0,45 | 0,66 |
| 8 | MSPs give equal value to local and scientific knowledge | 0,43 | -1 | 0,52 |
| 10 | MSPs focus mainly on environmental issues | -0,92 | 0,05 | -1,12 |
| 39 | MSPs are needed to negotiate trade-offs between different land users | 0,71 | 2,18 | 0,85 |
| 41 | MSPs do not improve collaborations among actors | -1,11 | -0,44 | -0,89 |
| 16 | MSPs can be a source of conflicts | -1,14 | -1,08 | -0,06 |
| 37 | MSPs are largely funded externally by the private sector, government, and donors | 1,12 | 0,8 | 1,6 |
| 18 | MSPs always recognize and prioritize local needs | 1,41 | 1,2 | 1,64 |
| 15 | MSPs facilitate the harmonization of policies and laws with traditional customs and regulations | 1,83 | 1,74 | 1,96 |
| 32 | MSPs fail to consider multifunctional landscapes | -0,57 | -0,51 | -0,74 |
| 42 | MSPs only exist where there is a project | -0,58 | -0,45 | -0,35 |
| 28 | MSPs only make sense if traditional authorities play important roles in them | 0 | 0,5 | -0,08 |
| 3 | MSPs don’t engage national policymakers | 0,14 | -0,62 | 0,03 |
| 5 | MSPs endeavor to facilitate the enforcement of charcoal regulations | 1,09 | 1,08 | 0,3 |
| 38 | MSPs only work if communities have a say in decision-making regarding natural resources and landscape | 1,17 | 0,03 | 1,18 |
| 17 | MSPs do not help increase communication across sectors | 0 | -1,84 | 0 |
| 23 | Only the private sector has the innovative capacity to manage landscape sustainability | 0 | -0,59 | 0 |
| 19 | MSPs hinder the adoption of best practices | 0 | -0,3 | 0 |
